# Experimental Consideration of Conditions for Measuring Residual Stresses of Rails Using Magnetic Barkhausen Noise Method

**DOI:** 10.3390/ma14185374

**Published:** 2021-09-17

**Authors:** Young-In Hwang, Yong-Il Kim, Dae-Cheol Seo, Mu-Kyung Seo, Woo-Sang Lee, Segon Kwon, Ki-Bok Kim

**Affiliations:** 1 Safety Measurement Institute, Korea Research Institute of Standards and Science, 267, Gajeong-ro, Yuseong-gu, Daejeon 34113, Korea; yihwang@kriss.re.kr (Y.-I.H.); yikim@kriss.re.kr (Y.-I.K.); dcseo@kriss.re.kr (D.-C.S.); 2 Department of Nano Science, University of Science and Technology, 217, Gajeong-ro, Yuseong-gu, Daejeon 34113, Korea; 3 Smart Control & Sensing, 187, Techno 2-ro, Yuseong-gu, Daejeon 34025, Korea; mukyung.seo@smartcs.co.kr (M.-K.S.); woosang.lee@smartcs.co.kr (W.-S.L.); 4 KORAIL Research Institute, Korea Railroad Corporation, 240, Joongang-ro, Dong-gu, Daejeon 34618, Korea; tibobkr@korail.com; 5 Department of Science of Measurement, University of Science and Technology, 217, Gajeong-ro, Yuseong-gu, Daejeon 34113, Korea

**Keywords:** non-destructive evaluation (NDE), magnetic method, magnetic Barkhausen noise, stress measurement, residual stress, railway rail

## Abstract

Residual stress, a factor affecting the fatigue and fracture characteristics of rails, is formed during the processes of fabrication and heat treatment, and is also generated by vertical loads on wheels due to the weight of vehicles. Moreover, damage to rails tends to accelerate due to the continuous increase in the number of passes and to the high speed of passing vehicles. Because this can have a direct effect on safety accidents, having a technique to evaluate and analyze the residual stresses in rails accurately is very important. In this study, stresses due to tensile loads applied to new rails and residual stresses remaining in used rails were measured by using magnetic Barkhausen noise method. First, a magnetization frequency and noise band suitable for the rails were selected. Moreover, by applying tensile loads to specimens and comparing the difference in magnetization amplitudes for each load, the stresses applied to the rails by using the magnetic Barkhausen noise method were measured, and the analysis of the results was verified. Based on these results, the difference in the results for the loads asymmetrically applied according to the wheel shape was analyzed by measuring for the head parts of used rails.

## 1. Introduction

Recently, in the railway field, the conditions during use of important parts, such as rails, wheels, and axles, have become increasingly harsh because the high-speed rail networks operating worldwide have increased. In the last decade, the length of the high-speed networks in commercial operation worldwide has increased by 3.6 times [1]. In particular, the rails are repeatedly loaded with extreme weight according to how the vehicles are operated, so defects in the rails can develop very quickly and lead to damages to the rails. Accordingly, under conditions in which rail defects cannot be detected early, the possibility of train-derailment accidents due to the rail damages increases. This means that reliability the evaluation of rails is essential to prevent such accidents.

The residual stresses remain inside materials after manufacture, processing, and heat treatment, which condition is known to cause destruction of structural materials [2]. Rails undergo a heat treatment process and a roller straightening process during manufacture, and at these times, residual stresses form inside [3,4]. In addition, the rails are subjected to vertical loads due to wheel rolling, to shear loads caused by train braking and traction during train driving, and to fatigue conditions due to repetitive loads [5,6]. When fatigue grows above a certain threshold, cracks grow under the influence of external stresses [7]. Therefore, the residual stress, which is a factor affecting the fatigue of the rails, is an important consideration when designing rails because it is closely related to their strength and lifetime.

Various approaches to measuring the residual stresses in rails have been studied. The simplest methods of measuring the residual stresses of the rails are the methods of destructive measurement. However, because it is practically impossible to cut rails in use, making measurements by using non-destructive methods is essential. Accordingly, a variety of methods have been tried for measuring residual stresses, involving such as neutrons, laser, magnetism, and ultrasonic waves [8,9]. Because non-destructive methods for the measurement of residual stresses in rails are highly demanding techniques, the effective methods of measurement with sufficient performance have not been established in Korea.

Maintenance decision-making is based on the experience of inspectors and the simple inspections of the surface condition of rails. Thus, effective and economical techniques for measuring the residual stresses in rails are required. The railways of advanced countries are already investigated and managed through non-destructive techniques for measuring residual stresses in various rails [10]. Nevertheless, it is necessary to develop suitable measurement techniques and management system because the management methods and operating systems that have been studied abroad do not fit the domestic situation. Moreover, because most studies have been conducted mainly on rails in other countries, there have been few studies of residual stresses involving the rail standards used in Korea.

The magnetic Barkhausen noise method measures voltage-type noises caused by sudden changes in the domain wall when changes in magnetization occur in ferromagnetic materials [11]. Magnetic Barkhausen noise has a close relationship with the microstructure of materials because it is related to the phenomenon that a magnetic domain wall suddenly deforms at locations such as grains, voids, and dislocations because discontinuous changes occur in magnetic flux [12,13]. In other words, by inferring the stress state of the structure by using the magnetic Barkhausen noise, it is possible to evaluate whether it is in a non-stressed state or in an inherent residual stress state. This technique has the advantage of being able to evaluate quantitatively residual stresses in the structure of ferromagnetic materials very simply and quickly. Accordingly, in recent years, the magnetic Barkhausen noise has been widely used in stress measurement. Yi et al. [14] investigated the effect of microstructure evolution on magnetic properties and properties of the magnetic Barkhausen noise during isothermal annealing for 12% Cr steel. Gauthier et al. [15] non-destructively measured the surface residual stress in structural steel samples with the magnetic Barkhausen noise method. Lachmann [16] employed the magnetic Barkhausen noise method to investigate the characterization of residual stress relaxation in fatigue loaded welded joints. Lindgren et al. [17] uniaxially strained mild steel specimens in tension at strain levels varying from 0.5 to 10%, followed by X-ray diffraction residual stress and the magnetic Barkhausen noise measurements. Moreover, they also evaluated the residual stresses of the ferrite phase of duplex stainless steels by the magnetic Barkhausen noise method [18]. Ju et al. [19] applied a modified magnetic Barkhausen noise method to obtain residual stress distribution in an API X65 pipeline weldment and different weldment microstructures affected magnetic response and yielded different magnetic Barkhausen noise values. Desvaux et al. [20] present a non-destructive method for estimating surface residual stress on several different batches of bearings in aeronautic engines, based on the magnetic Barkhausen noise effect. Stewart et al. [21] measured the magnetic Barkhausen noise for AS1548-7-460R steel in a pressure vessel under applied stress. Yelbay et al. [22] non-destructively determined residual stresses in welded steel plates by the magnetic Barkhausen noise method, and developed a stress calibration set-up with the magnetic Barkhausen noise and a residual stress measurement system with scanning ability. Mierczak et al. [23] studied the influence of elastic tensile and compressive stresses of various magnitudes on the magnetic Barkhausen emissions with the objective of developing a technique for quantitative measurements of surface stress in machined steels. Sorsa et al. [24] built a prediction model for residual stress and hardness of a case-hardened steel samples based on the magnetic Barkhausen noise measurements. Vourna et al. [25] determined residual stress distribution in welded non-oriented electrical steel samples through deformation measurements and appropriate math calculations. Ávila et al. [26] employed magnetic Barkhausen noise assessment for comparing the microstructural, hardness and residual stress evolution across a two-pass friction stir welding butt joint of a X80 pipeline steel. Dong et al. [27] employed the method of magnetic Barkhausen noise to achieve simultaneous, quantitative prediction of residual stress and surface hardness in box-shape deep drawn parts. Neslušan et al. [28] employed Barkhausen noise technique to non-destructive evaluation of rail way wheels across wheel width. Hizli et al. [29] investigated the applicability of the magnetic Barkhausen noise method to the measurement of surface residual stresses in the carburized steels. Liu et al. [30] applied a magnetic Barkhausen noise scanning test for evaluating the surface laser quenching induced changes in microstructure and residual stress. Srivastava et al. [31] assessed surface integrity, including metallurgical changes, surface roughness, and residual stress, using nondestructive magnetic Barkhausen noise and hysteresis loop technique. Oliveira et al. [32] investigated the magnetic and mechanical properties, as well as the surface residual stress on maraging steel 300 in three build orientations.

In this paper, a study on measuring the stresses on rails named 50 kgN [33], using the magnetic Barkhausen noise method, is described. First, a magnetizing frequency and a noise band suitable for inspecting the rails were selected. In order to compare magnetization amplitudes according to the difference in applied loads, the values of the magnetic Barkhausen noise were acquired while applying each tensile load. In addition, it was analyzed how the Barkhausen magnetic noise-intensity values differ according to the difference in loads applied asymmetrically to each part of the used rails. Through this study, it was verified that the residual stresses on the rails could be measured non-destructively by using the magnetic Barkhausen noise method.

## 2. Materials and Methods

Magnetic measurement technique is a non-destructive testing method that evaluates changes in magnetic properties among the physical properties of materials and verifies the safety of structures in which the materials are used. It has the advantage of being able to obtain data on many variables by measuring ferromagnetic materials. Magnetic variables that could be used to determine the intrinsic properties of materials include magnetizing force, magnetic flux density, coercivity, and permeability, which are closely related to the material’s crystal grain size, stress, plastic deformation, and precipitates.

Railway rails, the subject of this study, are made of ferromagnetic materials of which the major component is steel. The ferromagnetic material is not magnetized in its natural state, but when a strong magnetic field is applied from external, the magnetic moments are aligned in magnetic field direction and magnetized. Moreover, the magnetization remains even after the external magnetic field is removed as residual magnetization.

When an external magnetic field is applied to a ferromagnetic material having magnetic structures, the magnetic flux density of test object increases, and the magnetic domain wall moves. It is disturbed by retarding fields, such as various defects, including grain boundaries, dislocations, precipitates, nonmagnetic inclusions, dislocations, or voids. Moreover, a discontinuity can appear after which the domain wall jumps [11,12]. The force that appears at this time is called the retarding force, and the magnetic wall is moved discontinuously by this force. A pulse-like magnetic noise is generated that corresponds to rapid change in the magnetic flux. In this discontinuous magnetization process, the induced voltage that can be measured by using an induction coil is called magnetic Barkhausen noise [34]. When a ferromagnetic material is magnetized, a noise voltage is generated in coil wound around it, and when it is amplified, noise similar to a wave sound is generated in what is called the Barkhausen effect. Non-destructive testing techniques based on the magnetic Barkhausen noise can be used to determine microstructure factors or stress states in materials based on the magnetic behavior of ferromagnetic materials.

By understanding this effect, it is possible to understand the changes in the slope of hysteresis curve and the change in magnetic properties due to the change of constraining stress applied to rails according to loads. Basically, a magnetic Barkhausen noise measurement device is configured to measure magnetic Barkhausen noise signals from a detection sensor located on the surface of specimens by applying an alternating voltage after winding a magnetizing coil in a yoke. Depending on the stress state of the specimens, the movement state of a magnetic domain and magnetic walls changes, and the magnitude of the magnetic Barkhausen noise also changes. Accordingly, the parameters revealed by measuring magnetic Barkhausen noise generated through interaction between the magnetic structure and the microscopic and macroscopic structures of the object, provide relevant information about the state of the specimen.

## 3. Measurements

The test subject used to measure applied stresses is a rail called 50 kgN [33], which is used for freight and passenger transportation in Korea. Table 1 shows the material properties of the rail used in the experiments.

**Figure 1:**
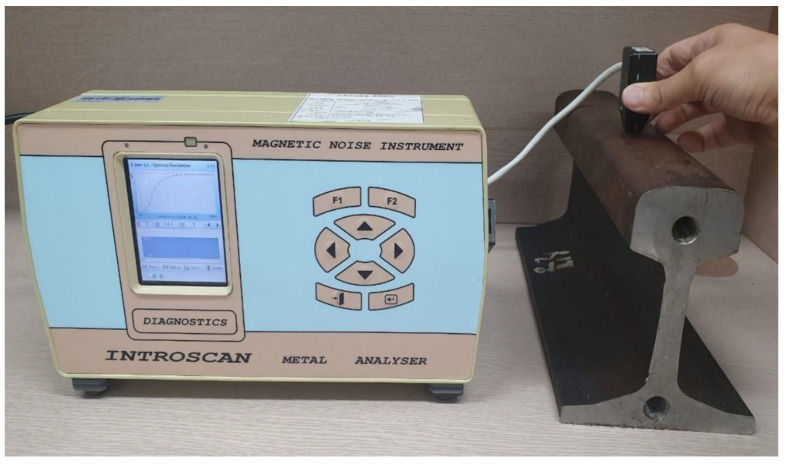
Photograph of the experimental setup for selecting experimental conditions to measure stresses on rails.

First, excitation amplitudes were measured by changing magnetic noise filtering range after fixing each specific test frequency to investigate changes in magnetic Barkhausen noise according to different magnetization frequencies and noise bands. A new rail that has never been used was employed as the test subject in the experiments, and signals were acquired at the head part of the rail. INTROSCAN, an analyzer developed by LLC “NPF Diagnostics” (Minsk, Belarus), was used as a magnetic Barkhausen noise measurement device [35]. The device is applicable to high-tech fields due to its high test-speed and inspection accuracy in calculating the properties of ferromagnetic materials. A sensor used included a magnetic yoke with a coil, and was driven by the generator of the analyzer. The ferrite sensor with a fine measuring coil between poles was placed to detect noise signals. The contact area of this sensor was 4.7 mm^2^. Magnetization frequencies selected for use in the experiments were 45, 60, 90, and 120 Hz, and magnetic noise filtering ranges were 3–15, 3–200, 3–1000, 20–200, 20–1000, and 200–1000 kHz. Figure 1 shows the photograph of the experimental setup for selecting set values to measure stresses on rails.

**Figure 2:**
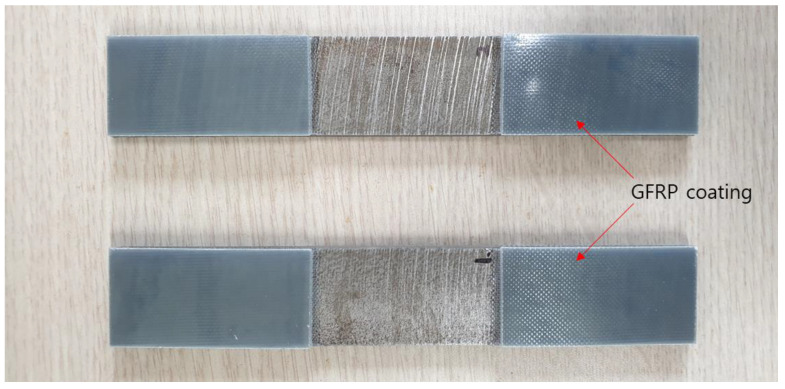
Photograph of specimens for experiment to measure stress under tensile loads.

Then, specimens for tensile test were extracted from the new rail. Water-jet cutting was chosen for manufacturing the specimens, because it minimizes the effects of stress disturbance due to rail cutting, compared to other processing methods. The dimensions of the specimens are 150 × 25 × 2.5 mm, and glass-fiber reinforced plastic (GFRP) was used to coat both ends of each specimen fabricated. It is because this prevents the jigs for tensile testing with electrical conductivity and the specimens from being directly attached. This eliminates the possibility that unwanted disturbance in electrical signals rather than magnetic Barkhausen noises could be entered into the experimental data. Figure 2 shows test pieces manufactured for use in the tensile test.

**Figure 3:**
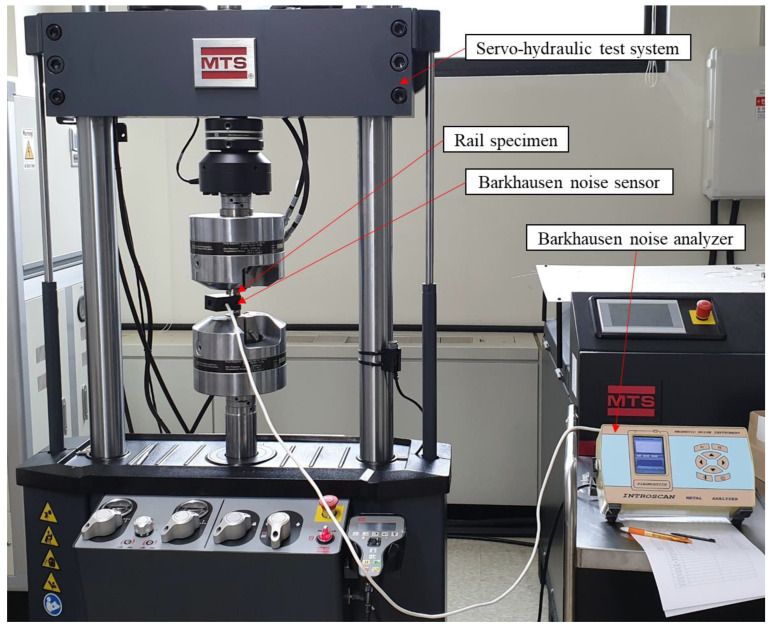
Photograph of the experimental setup for measuring stresses in rail specimens under tensile loads by the servo-hydraulic test system.

Each magnetic Barkhausen noise signal was measured while changing the applied loads by placing the magnetic Barkhausen noise sensor in the direction horizontal and vertical to the load direction. Because the magnetic Barkhausen noise signal changes according to the attachment force of the sensor, a jig was manufactured and used to maintain this adhesion force and thus minimize this variation. The same device was used to measure the magnetic Barkhausen noise and a servo-hydraulic test system (Landmark 370.10, MTS, Eden Prairie, MN, USA) used to apply the tensile force. Figure 3 shows the photograph of the experimental setup for measuring stresses in rail specimens under tensile loads.

**Figure 4:**
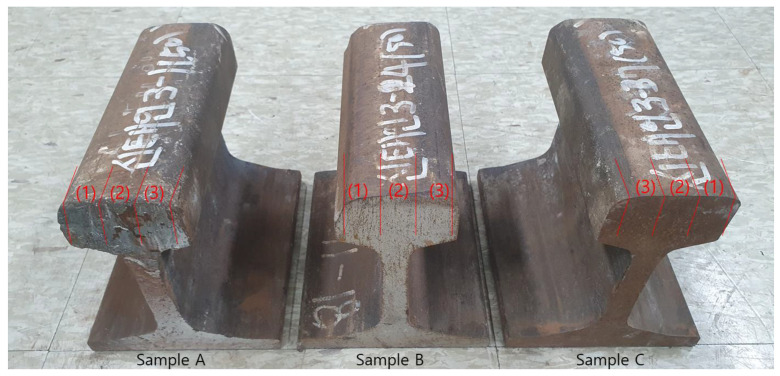
Photograph of three used rail specimens applied in experiments to measure residual stresses by magnetic Barkhausen noise.

At last, in order to conduct experiments using the magnetic Barkhausen noise on preloaded rails, three used rails were applied. Depending on the shape of wheels of railway vehicles, asymmetric loads were applied to the entire rails, because the wheels contact the upper surface of the rails. To analyze the differences according to response characteristics, using magnetic method, signals were acquired from three regions with the same cross-sectional area, as shown in Figure 4.

**Figure 5:**
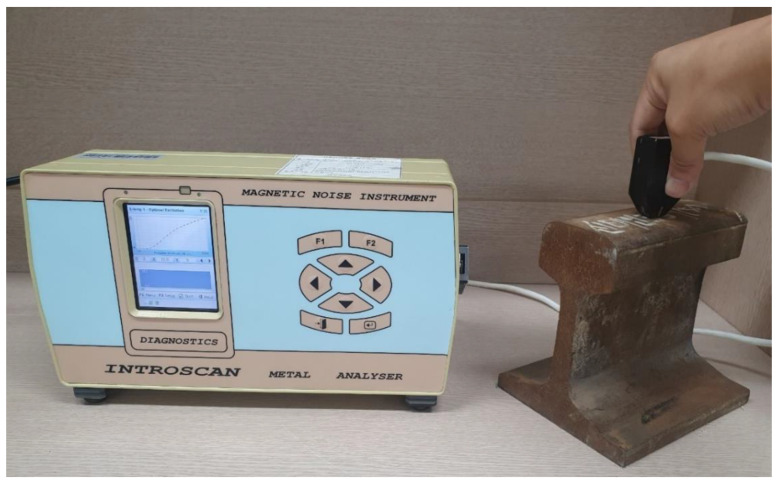
Photograph of the experimental setup for measuring residual stresses in the used rails.

In Region (1) (see Figure 4), there is a contact between the field side of the vehicle wheels and the rail, and there, the probability of contact is relatively lowest. In Region (2), contact occurs when the vehicle is driving on straight tracks or on curves with large radii. In Region (3), when driving on curves with small radii, contact between the wheels and the rail occurs, and due to the higher contact pressures and sliding velocities, severe wear occurs between wheel flanges and rail gauge corner. In this way, three measurement points were selected for each of the three rail test pieces, and experiment were performed for a total of nine positions. Figure 5 shows the photograph of the experimental setup for measuring residual stresses in the used rails.

## 4. Results

**Figure 6:**
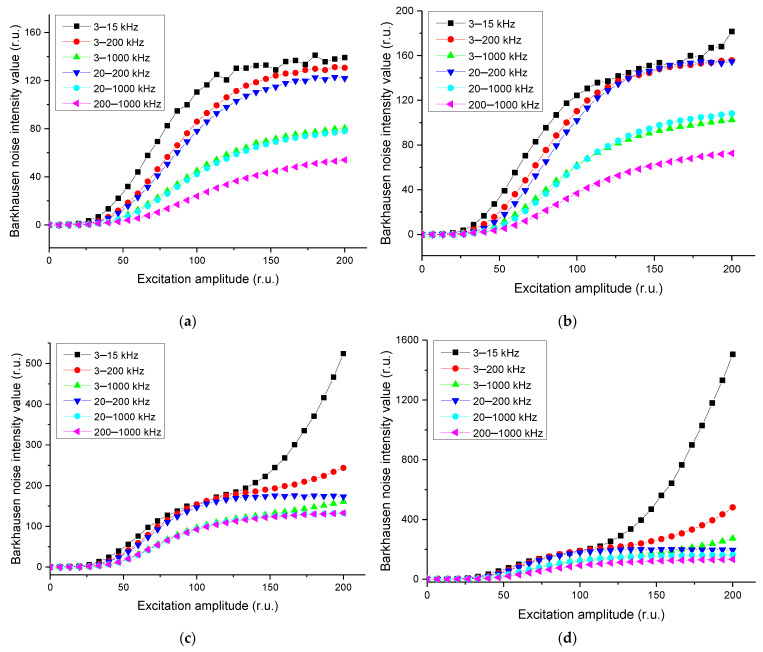
Measured magnetic Barkhausen noise intensity values for the indicated noise bands at magnetization frequencies of (**a**) 45 Hz, (**b**) 50 Hz, (**c**) 90 Hz, and (**d**) 120 Hz.

When the magnetization frequency was varied, magnetic Barkhausen noise intensity values were different for each noise band. The results are shown in Figure 6.

The intensity of the magnetic Barkhausen noise, displayed on the vertical axis, is a value proportional to the average value of the sensor’s the output voltage of the pickup coil for a specified number of magnetization reversal cycles. The number of these cycles is determined by a user-selectable measurement time constant. In practice, the system measures the induced voltage generated by Bloch wall jump [36,37], but the last registered amplitude value is the result of the absolute current incorporated into the internal circuit. Therefore, the magnetic Barkhausen noise intensity values are displayed in relative unit (r.u.). In principle, it can be converted to the induced electromotive force (EMF) of the pickup coil. However, for non-destructive testing, this does not make sense. This is because the amplitude in an arbitrary unit is related to mechanical stresses and calibration is required for determining the stresses.

Through this experiment, it was determined that the higher the applied magnetization frequency was, the higher the magnetic Barkhausen noise intensity values. Through this, the magnetization frequency of 120 Hz was determined to be the most suitable for inspecting changes of the magnetic Barkhausen noise according to residual stresses because signals with higher values could be obtained. In addition, 20–200 kHz, which showed high values with stable curves, was most suitable as a magnetic noise filtering range.

**Figure 7:**
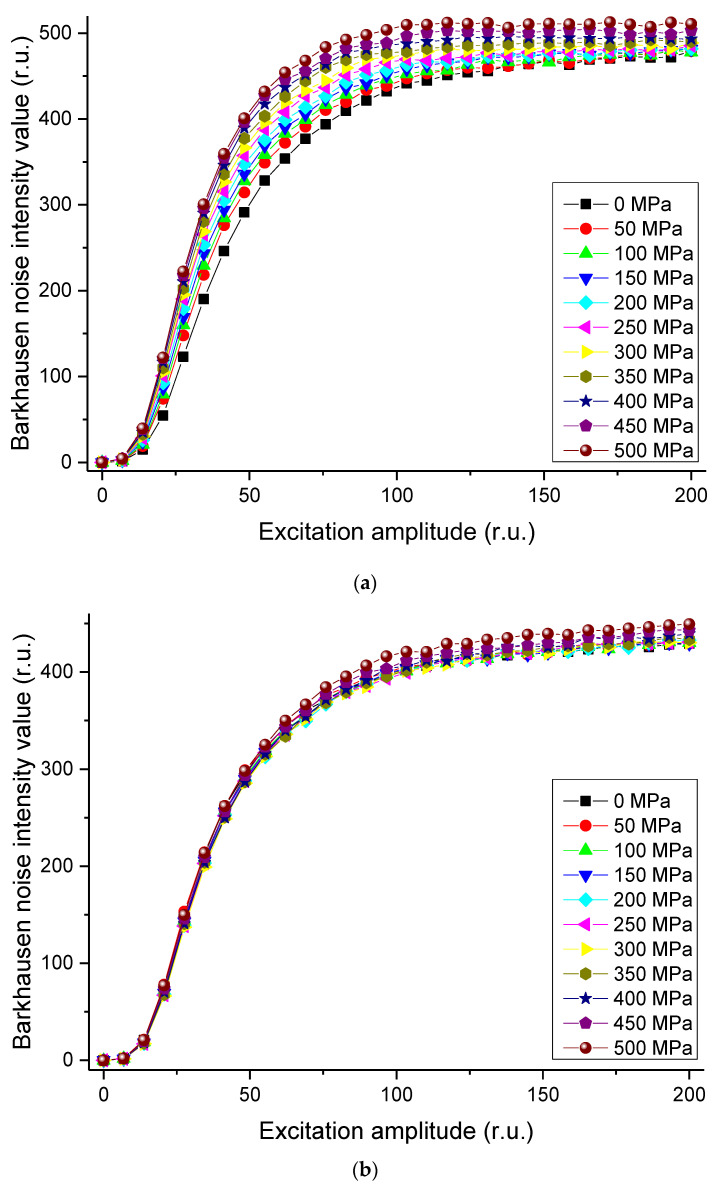
Measured magnetic Barkhausen noise intensity values for indicated tensile stresses in (**a**) longitudinal and (**b**) perpendicular directions.

The magnetic Barkhausen noise intensity values obtained with the sensor placed horizontally and perpendicular in the direction of the load while varying the applied load, using the selected magnetization frequency and noise band, are shown in the Figure 7.

Through this result, it was possible to verify the correlation between the magnetic Barkhausen noise signal and the magnetization amplitude obtained from tensile testing of the rail specimens. When the sensor direction was in line with the load direction, the magnetic Barkhausen noise signal increased in proportion to increase in the tensile load, and in particular, the magnetization amplitude showed a large increase within the range 25–100. However, within the range 100–200, the increase was not significant. On the other hand, when the sensor was in the direction perpendicular to the load direction, the signals did not show significant differences even when the tensile load increased. Through these results, it can be seen that magnetic Barkhausen noise patterns differ depending on the stress.

**Figure 8:**
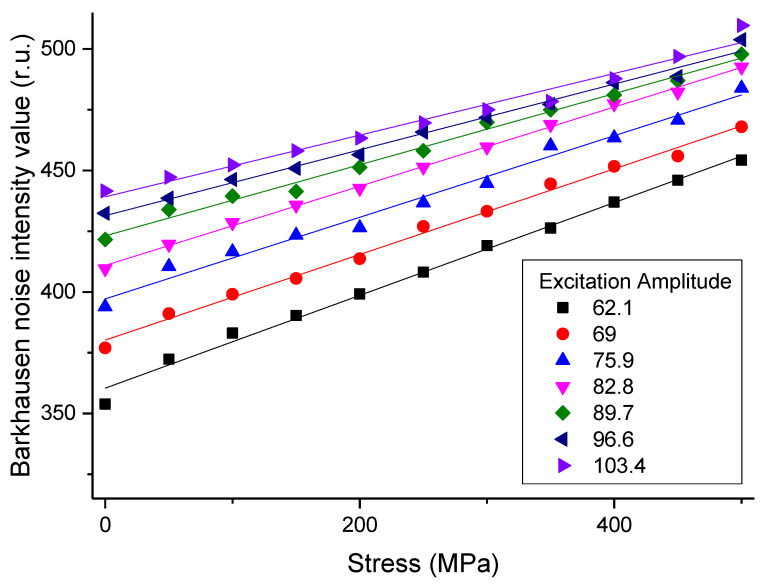
Result of calibration using regression analysis on stress in longitudinal direction vs. magnetic Barkhausen noise intensity value graph.

To verify that the results of this experiment were accurate, a calibration was performed by using regression analysis [38] on the stress vs. magnetic Barkhausen noise intensity value graph. The magnetic Barkhausen noise intensity values obtained by arranging the sensor in line with the load direction, are shown in Figure 8. These are the results of correlation analysis [39] performed by selecting excitation amplitude values that show the differences in the magnetic Barkhausen noise intensity values depending on the stress.

Table 2 shows the correlation coefficients according to the excitation amplitude values obtained through the graph above. For all selected excitation amplitudes, correlation coefficients converged to 1, and it was verified that the results obtained through this experiment were very reliable.

**Figure 9:**
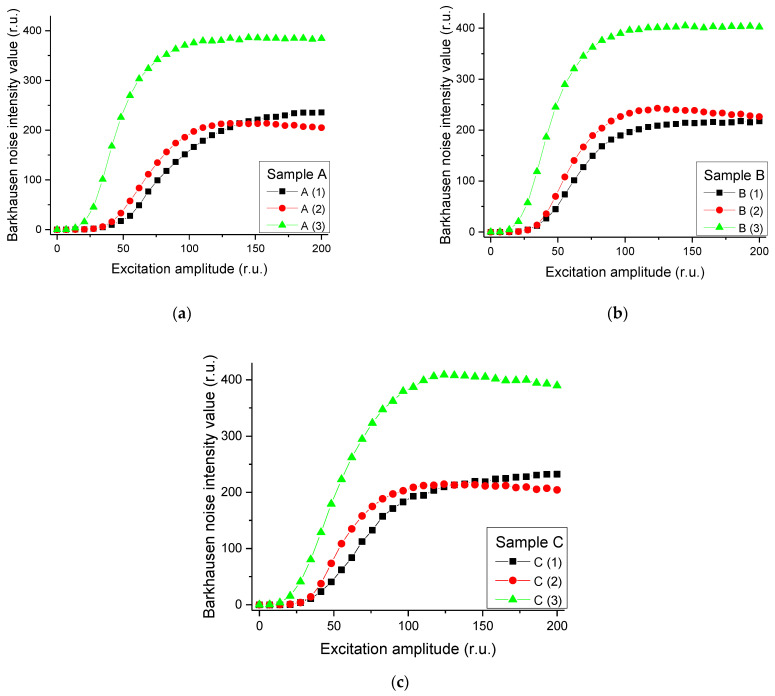
Measured magnetic Barkhausen noise intensity values with magnetization frequency of 120 Hz for Region (1) to (3) of each rail specimens: (**a**) Sample A, (**b**) Sample B, and (**c**) Sample C.

Excitation amplitudes were calculated for three rail specimens. The relationships between these values and the magnetic Barkhausen noise intensity values are shown in Figure 9 below.

Magnetic Barkhausen noise intensity values for the specimens from Region (3) (see Figure 9), where severe wear occurred due to high contact pressure and sliding speed, were found to be relatively higher than those from other specimens. Therefore, it was verified that the difference according to the residual stresses can be expressed by using magnetic Barkhausen noise.

## 5. Conclusions

The magnetic Barkhausen noise method was used to evaluate the stress of 50 kgN rails, using the magnetic properties of the material, and the following results were obtained through this study.

1. By comparing the experimental results while changing the conditions, a magnetization frequency and noise band suitable for 50 kgN rails were selected. The selected magnetization frequency was 120 Hz, and the selected noise band was 20–200 Hz.
2. It was found that the magnetic Barkhausen noise intensity values measured while applying tensile loads increased linearly with the increase in tensile load when the direction of the detection sensor was the same as the load direction. The magnetization amplitude showed a distinct tendency to increase within the range 25–100 of excitation amplitude.
3. When the direction of the detection sensor was perpendicular to the load direction, it was found that the magnetic Barkhausen noise signal hardly changed, even when the tensile load increased.
4. As a result of analyzing the correlation between the stress and the magnitude of the magnetic Barkhausen noise signal, it was found that the correlation coefficient was over 0.988. This indicates that stress can be measured with high reliability, using the magnetic Barkhausen noise method.

Through this study, it was possible to measure the stress applied to 50 kgN rails by using a technique that verifies that the magnetic Barkhausen noise is affected by residual or applied stresses in specimens. Moving forward, future studies can take advantage of these results to extend the analysis to different rail types. As a final remark, the development of portable equipment can be applied to employ this technique to actual sites and to measure residual stress in rails.

## Figures and Tables

**Table 1:** Mechanical properties of 50 kgN rail [33].

Yield Strength (MPa)	Modulus of Elasticity (MPa)	Linear Expansion Coefficient (mm/°C)	Elongation Percentage (%)	Hardness (HBW)
≥800	210,000	1.14 × 10^−5^	≥10	≥235

**Table 2:** Correlation coefficients between magnetic Barkhausen noise intensity value and stress at different excitation amplitudes of 120 Hz.

**Excitation Amplitude (r.u.)**	32.1	69.0	75.9	82.8	89.7	96.6	103.4
**Correlation Coefficient (R)**	0.996	0.997	0.992	0.999	0.995	0.995	0.988

## Data Availability

Not applicable.
